# Presence of Gastric Ulcers in Horses Used for Historical Races in Italy

**DOI:** 10.3390/ani14081247

**Published:** 2024-04-22

**Authors:** Sara Busechian, Simona Orvieto, Irene Nocera, Fabrizio Rueca

**Affiliations:** 1 Department of Veterinary Medicine, University of Perugia, 06126 Perugia, Italy; fabrizio.rueca@unipg.it; 2 Private Practitioner, 06100 Perugia, Italy; simona.orvieto@gmail.com; 3 Institute of Life Sciences, Sant’Anna School of Advanced Studies, 56127 Pisa, Italy; irene.nocera@santannapisa.it

**Keywords:** Equine Squamous Gastric Disease, Equine Glandular Gastric Disease, historical speed horseraces, jousting competitions

## Abstract

**Simple Summary:**

Gastric ulcers are quite common in horses with the highest prevalence found in racehorses. Historical horseraces are traditional horseriding competitions popular in Italy that can be divided into two different types: speed races and jousting tournaments. Anglo-Arabian and Thoroughbreds are used, with several restrictions present in the regulation of the specific competition, pertaining to the breed of the horse, training, and preparation for the competitions (i.e., clinical examinations, qualifiers). Management of the horses is quite similar to that of racehorses running on a track. The aim of this study was to determine the prevalence of gastric ulcers found in the glandular or squamous mucosa of 73 animals performing in historical horseraces and determine differences in disease presence and severity between type of competitions, training facilities and breeds of horses. The prevalence of lesions in both mucosae is quite similar to those found in horses performing flat races on a racetrack. Anglo-Arabians used for speed races are more affected than Thoroughbreds, probably because they are involved in a higher number of races and longer traveling times, which are all risk factors for lesions of the squamous and glandular mucosa. These findings highlight the need to implement therapeutic and prophylactic protocols against gastric ulcers also in horses involved in historical horseracing: feedings with a reduced amount of starch, hay nets to increase the amount of time spent eating, paddock turnout for most of the day and the use of supplements to support gastric health are effective measures to prevent the development of gastric ulcers. The use of omeprazole at therapeutic (4 mg/kg per os once daily) or prophylactic (1 mg/kg per os once daily) dosages can also be effective in periods of high stress, especially when horses travel for longer distance or are exercised or raced more. Further studies are needed in this population of animals to determine the differences and similarities with horses involved in racing on a track.

**Abstract:**

Equine Gastric Ulcers Syndrome (EGUS) is a worldwide disease present in equids of different breeds, activity levels, and age groups. It is divided into two different illnesses: Equine Squamous Gastric Disease (ESGD) affecting the squamous mucosa and Equine Glandular Gastric Disease (EGGD) affecting the glandular mucosa. The historical horserace is a traditional competition that is common in Italy. They can be divided into two different types: speed races and jousting tournaments. Anglo-Arabians and Thoroughbreds are used for the two competitions with training and management systems similar to those used in races performed on a racetrack. The aim of this study was to determine the prevalence of ESGD and EGGD in horses used for historical horseracing and evaluate the differences in the presence of the diseases in animals of the two different breeds used for the two types of competition. A cohort of 73 horses was enrolled in this study, which were stabled in 10 training facilities and performed two jousting tournaments and one speed race. An ESGD at least of grade 2 was found in 88% of horses with all degrees of severity seen; EGGD was diagnosed in 45% of animals. In this cohort of horses, the presence and severity of ESGD and EGGD are similar to that in reports in racehorses performing on racetracks. Anglo-Arabians used for speed races are more affected by ESGD and EGGD, which is probably because they are involved in a higher number of races and travel more during the year compared to Thoroughbreds used for jousting competitions.

## 1. Introduction

Equine Gastric Ulcer Syndrome (EGUS) is a worldwide disease of equids that is characterized by the presence of lesions of the stomach from hyperkeratosis to erosions to ulcers. A few years ago, a Consensus Statement from the European College of Equine Internal Medicine divided the disease in two based on the mucosa affected: Equine Squamous Gastric Disease (ESGD) with lesions in the squamous mucosa and Equine Glandular Gastric Disease (EGGD) if it affects the glandular mucosa [1,2]. Prevalence is variable in the equine population with the highest detected in racehorses, such as Thoroughbreds and Standardbreds, where it seems to increase the longer they are in training [1,2,3,4,5,6,7]: almost all animals in heavy training for racing showed evidence of the disease in several studies [1,2,3,4,5,6,7,8,9]. Management factors are implicated in the development of the disease, especially in Thoroughbreds: the days of heavy training during the week, the amount of grain fed and the number of meals per day, time since first brought to the current stabling facility, and the presence of stereotypic behaviours are all considered risk factors for the development of both ESGD and EGGD [1,2,3,4,6,8,10,11,12]. The first is caused by prolonged exposure of the squamous mucosa to gastric acids: the protective mechanisms of this lining (i.e., high electrical resistance, tight epithelial junctions and osmophilic phospholipid surfactant-like layer) are not able to sustain acid contact for long periods of time, and injury can occur with only 30 min of exposure [11]. The pathophysiology of EGGD is poorly understood at the moment, but it seems to be related to the breakdown of the normal defense mechanism of the glandular mucosa: prostaglandins especially seem to be a key factor in the protection of the lining from acid injury, regulating blood flow, mucus and bicarbonate secretion and acid production. Other mechanisms involved could be disruption of the hydrophobic surface of the mucosa, changes in the bacterial microbiome or stress and response to it [12]. Clinical signs are similar for both diseases with poor body condition score, ill thrift, recurrent colic and poor performance being the most commonly described. A high percentage of horses can show no symptoms, and a correlation between signs and severity of the disease has not yet been found [1,2,4,8,11,12]. Diagnosis is based on gastroscopic examination of the stomach, and different grading scores have been proposed: for ESGD, the one proposed by the ECEIM Consensus Statement is considered the most reproducible, and it is the most commonly used [1]. For EGGD, on the other hand, no scoring system has been yet validated, and lesions should be described based on their macroscopic appearance [1,2]. Treatment is based on the use of gastroprotectant drugs, such as omeprazole, which for EGGD is associated with sucralfate [1,2,4,8,11,12]. For lesions of the glandular mucosa, due to their poor response to antiacid drugs, the use of misoprostol has been advocated [4,8,12,13]. Prognosis is good for healing of the lesions with better response to treatment found for ESGD compared to EGGD [8,14]. Recurrence is common, though, and changes in management are warranted whenever possible, especially in feeding regimens (reducing the amount of starch in the diet, feeding hay ad libitum during the day, etc.) and management (paddock turnout for at least some time during the day, reducing the time spent exercising per week) [4,8,11,12,15].

Historical horse races are a traditional type of horse racing present in different areas of Italy, where riders dressed in historical garb ride horses around a circuit. They can be divided into two types: speed races and jousting tournaments. The former are flat horse races usually performed around a square or an historical location, and only Anglo-Arabians can be employed: the most famous is “Palio di Siena” [16]. During jousting tournaments [17], riders run around a circuit with a lance on their hand, and they need to either collect a bounty or touch the shield of a dummy without being hit by its recoil; both the time needed to finish the circuit and the completion of the task are factors to determine the winner of the tournament. In this last type, no breed restriction is present, and Thoroughbreds are usually employed because of their speed. In both types of races, rules are in place to prevent injury to both animals and riders, and track conditions and surfaces are strictly regulated [18,19]. The breeds of horses used are similar to those exercised on racetracks, and most of the subjects employed are either chosen from those retired from the racetrack or used for both types of competitions (historical and racing on a track) at the same time.

No information is available on the effect of training for historical races on the gastric health of the horses involved in these competitions. The aim of this study was to detect the prevalence of gastric ulcers in a group of animals used for these competitions and determine if the type of competition (race or jousting) can influence the presence of ESGD and EGGD.

## 2. Materials and Methods

### 2.1. Horses

A cohort of horses used for historical horse races in Italy was enrolled in the study. All animals were in training for either speed races or for jousting tournaments at the time of the endoscopy and were housed in 10 training facilities. The examination was part of a routine checkup of the horses at the beginning of the year and was performed at the request of the referring veterinarian or the owner; oral informed consent was obtained from the owner. Before the gastroscopy, information about the breed and percentage of Thoroughbred blood in the pedigree, sex, age, name of the race they were mainly prepared for, any horseracing performed outside of the historical circuit and concurrent treatments were collected from the owner, trainer, or rider. Questions about management and training strategies were also part of the initial interview (Table 1). Any animal, irrespective of breed, age or sex was included, while treatments with any drugs or signs of systemic diseases were considered criteria for exclusion. To maintain anonymity, training facilities were recorded using a capital letter (i.e., A, B, C).

### 2.2. Gastroscopic Examination

Gastroscopy was performed according to the literature [1,2,20], using a 3 m long scope (60130PKS, Karl Storz Endoscopy, Tuttlingen, Germany) and a portable processor (Tele Vet X Led, Karl Storz Endoscopy, Tuttlingen, Germany). Feed and water were withheld for at least 16 and 4 h, respectively. After clinical examination, the horses were sedated with xylazine (0.25–1.1 mg/kg IV) and contained with a twitch. The scope was passed through the ventral nasal meatus and the upper respiratory tract toward the cranial esophageal sphincter and through the organ to the stomach. Here, the mucosa was examined completely in all parts, dilating the lumen with air, and cleaning the surface with water whenever necessary. The video was recorded and stored. Lesions of the squamous mucosa were graded according to the literature, while, due to a lack of a validated scoring system, those on the glandular one were only described. For statistical purposes, horses were considered positive for EGGD with any alteration of the mucosa, and they were considered positive for ESGD for grades of at least 2 [1,2,10].

### 2.3. Statistical Analysis

Information about the signalment of the horses (age, breed, sex, percentage of Thoroughbred blood in their pedigree), type of race they were involved in, training facility they were stabled at and any racing outside of the historic one they performed were collected on an Excel sheet and used for descriptive statistics. The presence and grades of ESGD and EGGD were also recorded. Normality was tested with a Shapiro–Wilk test. A Spearman test was used to evaluate the correlation between age of the horses and percentage of Thoroughbred blood and presence of ESGD, EGGD and both diseases at the same time. The same test was used to check the correlation between the same parameters and grade of ESGD. Wilcoxon and Kruskal–Wallis rank sum tests were used to assess differences in the presence and severity of the diseases between groups of horses divided by breed, sex, races other than historical ones, type of the historical horse race and training facility. Significance for all tests was set at *p* < 0.05. Statistical analysis was performed using “RStudio 2023.12.1+402” [21].

## 3. Results

### 3.1. Horses

Seventy-three horses were enrolled in the study, which were aged between 3 and 15 years (mean 7, median 6, interquartile range 3–15 years); 44/73 (60%) were Thoroughbred and 29/73 (40%) Anglo-Arabians; 32/73 (44%) were geldings, 37/73 (51%) were females and 4/73 (5%) were males. The horses enrolled were mainly trained for three historical horse races: Palio di Siena (28/73, 38%), Giostra dell’anello di Narni (38/73, 52%) and Giostra della Quintana di Foligno (7/73, 10%). The first historical race is a speed race, while the others are jousting tournaments. Ten training facilities were involved, all preparing the horses for a specific race: the distribution of the animals in the different stables and type of historical competition can be found in Table 2.

Overall, 9/73 animals (12%), all Anglo-Arabians trained for Siena, were also involved in flat races in racetracks. All horses were trained at least 6 days a week for a minimum of 30 min, mainly for speed, but at least once a week, the facilities preparing for jousting included also training specific for the race they were involved in. Management also was similar: all animals were housed in boxes, with straw as bedding, with minimal paddock turnout for some hours during the week. They were fed hay and grain twice a day: quantities were different in the facilities, but between 10 and 12 kg of hay (around 2–2.5 kg/100 kg BW) and 4 and 6 kg of grain (around 1–1.5 kg/100 kg BW) were given to each horse, depending on the body weight estimated by each trainer. Races had not yet started for the season, but the animals were in training throughout the wintertime. No subjects, except two (one trained in C and one in F), showed signs of gastric ulcers: the two symptomatic horses presented poor body condition score (3/9) and recurrent colic signs (pawing, laying down and poor appetite once a month for the last few months) that lasted for a short time and did not require treatment but resolved on their own. These horses were evaluated by the referring veterinarian, and gastroscopy was part of the diagnostic plan.

### 3.2. Gastroscopic Examination

**Figure 1:**
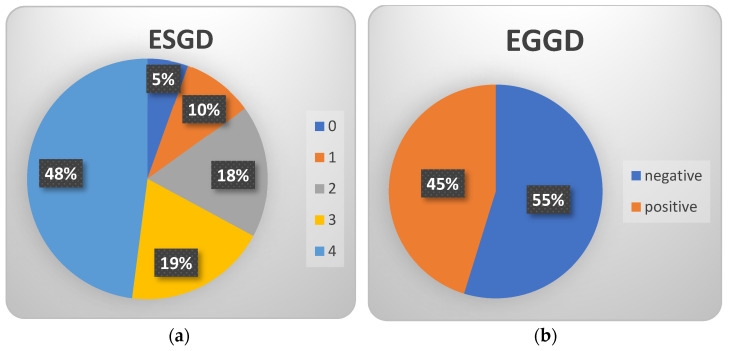
Prevalence of ESGD and EGGD in this population of 73 horses used for historical horseracing. (**a**) Percentage of the different grades of ESGD: grade 0/4: 4/73, 5%; grade 1/4: 7/73 (10%); grade 2/4: 13/73, 18%; grade 3/4: 14/73, 19%; grade 4/4: 35/73, 48% (**b**) Presence of EGGD: positive 33/73, 45%, negative 40/73, 55%.

ESGD was detected in 62/73 (88%) of horses: grade 2 was present in 13/73 (18%), grade 3 in 14/73 (19%) and grade 4 in 35/73 (48%); 11/73 (15%) were negative for ESGD, with 4/73 (5%) presenting grade 0 and 7/73 (10%) grade 1. EGGD was diagnosed in 33/73 (45%) of animals, as flattened areas of hyperemia within the glandular mucosa in different areas of the stomach body; erosions or bleeding ulcers were not seen in any of the horses examined. ESGD and EGGD were present in 18/73 (52%) subjects at the same time (Figure 1). Together with the referring veterinarian, treatment and management plans were proposed to the owners of all horses positive for ESGD and EGGD.

### 3.3. Results of the Statistical Analysis

Considering ESGD, its presence was correlated with Palio type (*p* = 0.03), while its severity was associated with breed (*p* = 0.02), percentage of Thoroughbred blood in the pedigree (*p* = 0.02), racing outside of the historical ones (*p* = 0.03), type of the competition (*p* = 0.004) they were trained for, and facility they were prepared at (*p* = 0.002). The presence of EGGD was associated with type (*p* = 0.03) and name (*p* = 0.02) of the competition horses were trained for and facility they were prepared at (*p* = 0.02). ESGD and EGGD diagnosed at the same time were correlated with breed (*p* = 0.02), percentage of Thoroughbred blood (*p* = 0.02), name (*p* = 0.01) and type (*p* = 0.01) of competition and training facility (*p* = 0.01). Results of the statistical analysis can be found in Table 3.

## 4. Discussion

EGUS is a condition of horses recorded in different breeds, age groups and exercise activities, with the highest prevalence found in heavy exercising animals, such as racehorses. In our population, the prevalence of ESGD is similar to reports from racetracks, where it can be up to 90% and more in older horses that have been longer in training [1,6,8,11]. All grades of ESGD were recorded with most of the horses presenting the highest severity: this is also similar to reports from racetracks [1,4,8,11]. The presence of EGGD is also in line with current literature findings, where between 25 and 65% of Thoroughbred are diagnosed with the disease [1,2,4,5,7,8,9,12]. The reason for this similarity can be found in the management system used: all animals in this study presented risk factors for ESGD and EGGD. They were trained for at least 6 days a week, and the amount of grain fed was similar to that usually given to racehorses, because the intensity of the training could be considered comparable [1,2,3,4,8,10,12,22,23].

Age is not a risk factor in this population, but all horses had been in training for at least a year either for historical races or those on a racetrack before the gastroscopy. Exercise intensity and duration have been considered risk factor for lesions in both mucosae of the stomach, because of the changes in abdominal pressure and exposure of the squamous lining to the acidic gastric content secondary to the movement, and increases in gastrin levels have also been recorded after an effort [24,25]. Training at least 6 days a week, as was the case also in this cohort of horses, has been associated with an increased presence of EGGD, while a lower severity of ESGD has been seen in horses performing less than 5 days per week [3,10]. Sex is not related to ESGD or EGGD in this group, which was indicated also in the literature [1,2,4,8,11,12].

In this population, the breed and percentage of Thoroughbred blood in the pedigree of the horses was associated with the severity of ESGD and with the presence of EGGD and ESGD at the same time. Despite the similar training and management conditions, Anglo-Arabians, which in this study seem more affected by the diseases, are used for both historical races and those on a racetrack, and they tend to travel more compared to horses used for jousting tournaments. These findings are confirmed by the correlation between presence and severity of the diseases and the type of competition (jousting or speed) and training facility. All historical horseraces are strictly regulated with rules pertaining to the type of track, the welfare of horses and riders involved and especially the breeds of animals that can be employed: in speed races, only Anglo-Arabians can be employed, while no restrictions are given for subjects involved in jousting [16,17,18,19]. Despite the fact that in this study, racing outside of the historical circuit has been associated only with the severity of ESGD, traveling has been considered a risk factor for the development of lesions in both mucosae [1,2,3,4,8]. Horses involved in speed races are used for different tournaments all throughout Italy, while those trained for jousting tend to race in competitions only in central Italy, and their schedule is less busy than the others. Training facilities were found to determine the severity of ESGD and presence of EGGD as well as both ESGD and EGGD in the same horse. Training intensity, management and feed (considering the amount of grain and hay fed per 100 kg of estimated body weight) were similar in the different facilities, and the only difference was the type of breed trained and the type of competition: Anglo-Arabians were racing more and traveling for a longer time. Horses used for speed races are used for competitions both in central Italy and in the north, racing more or less once a month in historical races, and traveling for up to 5 h once every two to three months. On the other hand, animals used for jousting compete only in central Italy, racing once a month or less, and traveling for 2 to 3 h once every couple of months or less.

Currently, no validated grading system for EGGD is described in the literature, so comparing the severity of the disease between studies is difficult. Reading the descriptions of the lesions in Thoroughbred from other publications [2,3,4,12], though, it appears that our population experiences less severe alterations, with only areas of hyperemia without erosions or ulcers identified. The clinical importance of the lesions of the glandular mucosa is not currently defined [2,8], so no information is available about the effect that different severities could have on clinical signs. It needs to be considered, though, that clinical signs in this population were present only in two horses with poor body condition score and recurrent colic: both were positive for EGGD. The trainer did not report reduced performance or girthiness (both related to ESGD and EGGD) [1,2,4,8,11,12,26,27].

The study had several limitations. Firstly, the horses were selected by referring vets in agreement with owners and/or trainers, and not all those present in the training facility were investigated. It is not possible to exclude, therefore, that despite not reporting clinical signs, the animals were selected based on their importance to the facility or to other criteria not disclosed by the trainers. Secondly, the number of horses enrolled per facility is different (Table 2), and this could influence the results of the statistical analysis: it needs to be considered, though, that the management is similar in all the stables both from a training and a diet point of view, and these seem to be the main risk factors for the development of gastric ulcers.

## 5. Conclusions

Horses involved in historical horse races in Italy show a similar prevalence of ESGD and EGGD than racehorses on racetracks: the type of training and level of exercise seems to be similar to those of “conventional” horse racing with a similar effect on the gastric mucosa. The presence and severity of ESGD and EGGD in this population are related to the breed and the type of competition the horses are involved in: Anglo-Arabians used for speed races seem to be more affected by both ESGD and EGGD together, and this could be related to the more intense training season that these horses experience, being involved in races in both northern and central Italy, with longer time traveling compared to Thoroughbred horses used for jousting competitions.

## Figures and Tables

**Table 1:** Questionnaire administered to owners, trainers, and riders during the initial interview.

Name of the horse Sex Age Percentage of Thoroughbred blood in the pedigree Name of the historical horserace the horse is trained for Does your horse race also on a racetrack? Is your horse stabled in a box? What kind of bedding do you use? Is your horse stabled in a paddock? For how long during the day? How many days a week? How many times a day do you give hay to your horse? How much? Do you train your horse specifically for the jousting tournament? How many times a week? Did your horse show signs of gastric ulcers (i.e., reduced performance, recurrent colic, poor body condition score, girthiness, etc.)? Please describe them

**Table 2:** Horse distribution according to type of historical competition and training facility.

**Type of Competition**	**Training Facility**	**Number of Horses (Percentage)**
Speed race	A	17/73 (23%)
	B	5/73 (7%)
	C	1/73 (1%)
	I	4/73 (5%)
	J	1/73 (1%)
Jousting competition	D	14/73 (19%)
	E	14/73 (19%)
	H	10/73 (14%)
	F	5/73 (7%)
	G	2/73 (3%)

**Table 3:** Results of the statistical analysis.

**Disease**	**Parameter**	***p* Value**
Presence of ESGD	Palio type	0.03
Severity of ESGD	Breed	0.02
	Percentage of Thoroughbred blood in the pedigree	0.02
	Racing outside of the historical circuit	0.03
	Type of competition (speed or jousting)	0.004
	Training facility	0.002
Presence of EGGD	Type of competition (speed or jousting)	0.03
	Name of the competition	0.02
	Training facility	0.02
Presence of ESGD and EGGD at the same time	Breed	0.02
	Percentage of Thoroughbred in the pedigree	0.02
	Type of competition (speed or jousting)	0.01
	Name of the competition	0.01
	Training facility	0.01

## Data Availability

Dataset available on request from the authors.
